# Increased Expression of X-Linked Genes in Mammals Is Associated with a Higher Stability of Transcripts and an Increased Ribosome Density

**DOI:** 10.1093/gbe/evv054

**Published:** 2015-03-18

**Authors:** Marie-Line Faucillion, Jan Larsson

**Affiliations:** Department of Molecular Biology, Umeå University, Sweden

**Keywords:** dosage compensation, RNA stability, sex chromosomes, RNA half-life, ribosome density

## Abstract

Mammalian sex chromosomes evolved from the degeneration of one homolog of a pair of ancestral autosomes, the proto-Y. This resulted in a gene dose imbalance that is believed to be restored (partially or fully) through upregulation of gene expression from the single active X-chromosome in both sexes by a dosage compensatory mechanism. We analyzed multiple genome-wide RNA stability data sets and found significantly longer average half-lives for X-chromosome transcripts than for autosomal transcripts in various human cell lines, both male and female, and in mice. Analysis of ribosome profiling data shows that ribosome density is higher on X-chromosome transcripts than on autosomal transcripts in both humans and mice, suggesting that the higher stability is causally linked to a higher translation rate. Our results and observations are in accordance with a dosage compensatory upregulation of expressed X-linked genes. We therefore propose that differential mRNA stability and translation rates of the autosomes and sex chromosomes contribute to an evolutionarily conserved dosage compensation mechanism in mammals.

## Introduction

Evolution of sex chromosomes from ancestral pairs of autosomes to dimorphic sex chromosomes leads to gender differences in gene dose. Although some genes located on the X-chromosome are expressed in a sex-specific manner, most genes require equal expression in males and females (Vicoso and Bachtrog 2009; Stenberg and Larsson 2011; Mank 2013). The mechanisms maintaining gene expression balance form part of the dosage compensation system, which must equalize expression levels between the two X-chromosomes in the homogametic sex and the single X-chromosome in the heterogametic sex, as well as balancing relative expression levels between the sex chromosomes and the autosomes. One model for this equalization of gene expression is Ohno’s hypothesis (Ohno 1967), which proposes a “doubling [of] the rate of product output” from the X-chromosome in both sexes to compensate for the degeneration of the proto-Y chromosome homolog. The resulting overexpression in females is believed to have driven the evolution of X-inactivation in mammals.

Ohno’s hypothesis has been tested, in most cases with the assumption that the average expression level between the X-chromosome and the autosomes, the X:AA ratio, ranges from 0.5 (no compensation) to 1 (full compensation). A close to 2-fold increase in expression of the X-chromosome is supported by a number of studies (Gupta et al. 2006; Nguyen and Disteche 2006; Yildirim et al. 2012), but has recently been challenged by claims based on RNA-sequencing (RNA-seq) analyses that there is no global increase in X-chromosomal gene expression (Xiong et al. 2010). However, several authors who have reanalyzed the RNA-seq data have drawn the opposite conclusion (Deng et al. 2011; Kharchenko et al. 2011; Lin et al. 2011). The controversy is based on disagreements about whether or not weakly expressed genes should be included in the analysis, as results of expression data analysis are very sensitive to the filtering method used, and unexpressed or weakly expressed genes can introduce more bias and noise than robust data (Castagne et al. 2011). An alternative approach is to compare expression of orthologous genes in species that separated before the formation of mammalian sex chromosomes, thereby comparing expression gene by gene with no need to assume similar expression levels between the X-chromosome and the autosomes. Using this approach, Julien et al. (2012) and Lin et al. (2012) reported that, for most genes studied, X-chromosome gene expression halved as the Y-chromosome degenerated during evolution. Another possibility is that dosage compensation is not complete in humans and only dosage-sensitive genes (most likely genes encoding proteins that are components of large complexes) need to be compensated (Pessia et al. 2012). Although most studies in mammalian systems have focused on determining the presence of global upregulation, rather than potential mechanisms, a recent article has suggested that the increased expression of mammalian X-chromosome genes is a combined effect of enhanced transcription initiation, increased RNA half-life, and MOF-mediated histone 4 lysine 16 acetylation (H4K16ac) of the male X-chromosome, which enhances chromatin accessibility and thus transcription output (Deng et al. 2013). See Birchler (2012) and Pessia et al. (2013) for reviews of this debate.

In fruit flies, dosage compensation involves a 2-fold increase in expression of male X-chromosome genes (Prestel et al. 2010; Stenberg and Larsson 2011), supporting Ohno’s hypothesis. This involves a combination of general buffering effects that act on all monosomic regions (Stenberg et al. 2009; Zhang et al. 2010; Lundberg et al. 2012) and the specific targeting and stimulation of the male X-chromosome by the male-specific lethal (MSL) complex. The MSL-complex consists of five proteins (MSL1, MSL2, MSL3, MLE, and MOF) and two redundant long noncoding RNAs (*roX1* and *roX2*) (Gelbart and Kuroda 2009; Prestel et al. 2010; Conrad and Akhtar 2011) which are essential for correct targeting of the MSL-complex to the X-chromosome (Figueiredo et al. 2014). The upregulation of the male X-chromosome is believed to be partly due to the enrichment of H4K16ac catalyzed by the acetyltransferase MOF. Although the increased expression of X-linked genes in male flies is generally accepted, the mechanisms involved have not been elucidated. Whether the MSL-complex-mediated increased expression is due to increased, transcriptional elongation (Larschan et al. 2011; Prabhakaran and Kelley 2012) or initiation (Conrad et al. 2012; Vaquerizas et al. 2013) is hotly debated (Ferrari et al. 2013, 2014; Straub and Becker 2013). 

In the study presented here, we adopted a new approach to investigate dosage compensation in mammals, in which a 2-fold general increase in expression of X-chromosomes is not widely accepted. We hypothesized that progressive degeneration of the proto Y-chromosome must have led to evolutionary pressure at all levels to compensate for losses of functional gene copies. Thus, compensation may occur through several mechanisms acting at distinct regulatory steps of gene expression (transcription, RNA stability, translation, and protein stability). Although most previous studies have focused on the first step, transcription, we have examined the potential contributions of mRNA stability and translation rates to dosage compensation. More specifically, we have analyzed differences in RNA decay and ribosome densities of transcripts of the X-chromosome and autosomes to determine whether dosage compensation may be partly accomplished through global control of RNA stability and translational activity of the X-chromosome. Our results show that X-chromosome transcripts have significantly longer half-lives than those of autosomal genes in both human males and females, and suggest that it could be conserved across mammals. Furthermore, using coupled human and mice ribosome sequencing (ribo-seq) and RNA-seq data sets, we show that X-chromosome transcripts have a higher ribosome density than autosomes, which may both lead to higher protein production and account for the observed increase in mRNA stability.

## Materials and Methods

### BRIC-seq Analysis of Half-Lives of Transcripts from Each Chromosome in HeLa Cells

To compare half-lives of X-chromosome and autosomal transcripts, we first used half-life data for transcripts of 11,679 genes (including 353 located on the X-chromosome) obtained by (Tani, Imamachi, et al. 2012). All genes for which transcript half-lives had been calculated were included in the analysis. Mean and median mRNA half-lives, mean reads per kilobase per million mapped reads (RPKM) values, and mean mRNA lengths were calculated for each chromosome individually and the whole set of autosomes. Housekeeping and nonhousekeeping genes were segregated according to the classification by Zhu et al. (2008). The examined set included 5,702 and 171 housekeeping genes, together with 5,624 and 182 nonhousekeeping genes, on the autosomes and X-chromosome, respectively. To assess spatial distributions of average mRNA half-lives of genes along the chromosomes, the genes of each chromosome were ordered according to their coordinates. The p and q chromosome arms were then separated and the average half-life of transcripts of genes along each chromosome was calculated using a sliding window of 30 genes moving one gene per step. The bin in which the X-chromosome inactivation center (*Xic*) is situated in the middle was localized using the closest gene available, that is, *RLIM*.

### Analysis of Human HapMap Lymphoblastoid Cell Line Data Sets

We analyzed preprocessed data on the stability of transcripts in seven human lymphoblastoid cell lines (LCLs) published by Duan et al. (2013). Average half-lives of transcripts of genes on each chromosome in each cell line were computed. Male cell lines and female cells lines were averaged independently. When replicate data were available they were averaged. The analysis covered 10,951 genes (in all replicates), 317 of which were present on the X-chromosome and no cutoffs were applied.

### Analysis of mRNA Half-Lives in Human B Cell and Murine Fibroblast Data Sets

Data were downloaded from supplementary files published by Friedel et al. (2009) and the average half-life of mRNA expressed from each chromosome was compiled using precalculated mRNA half-life values. The analysis covered 7,923 and 4,873 genes in human B cells and murine fibroblasts, respectively, including 265 and 151 X-chromosome genes, respectively.

### Correlations between Human mRNA Half-Life Data Sets

To calculate correlations between human mRNA half-life data sets, only genes for which stability data were available in all data sets were used. This included 5,374 genes in total, 158 of which were on the X-chromosome.

### Ribo-seq Analysis Using HeLa Cell and Mouse Neutrophil Data Sets

To probe interchromosome variations in ribosome densities of transcripts, HeLa cell and mouse neutrophil data sets processed by Guo et al. (2010) were downloaded from GEO database (accession numbers: GSM546920, GSM546921, GSM546926, GSM546927, GSM546987, and GSM546988). Genes were mapped to their respective chromosomes using Bioconductor annotation databases against the refseq ID provided or the gene name. Cutoffs were applied to filter out low read count and low RPKM entries (ribo-seq read count > 10, RNA-seq read count > 50, ribo-seq RPKM > 2, RNA-seq RPKM > 5). The remaining histone genes (poly(A) minus) were also removed from the data set because their actual RNA levels cannot be determined using a poly(A) selection protocol. The ribosome density for transcripts of each gene was calculated as the ratio between the RPKMs obtained in the ribo-seq and RNA-seq experiments. Data obtained from the BRIC-seq analysis of HeLa cells and RNA stability in fibroblasts analysis (described earlier) were then, respectively, used to explore correlations in humans and mice between these ratios and average mRNA half-lives of transcripts from each chromosome. All the summary statistics were calculated using custom R scripts.

### Analysis of Average Chromosomal Poly(A) Tail Length

To analyze interchromosome variations in transcripts’ poly(A) tail lengths, TAIL-seq data from HeLa cells were downloaded from supplementary file S1 published by Chang et al. (2014). The data set was merged with the mRNA half-life data from the BRIC-seq HeLa cell data set, with chromosomal assortment. Entries with incomplete data were discarded (1,122). The combined analysis included 3,870 genes, 135 of which were located on the X-chromosome. The arithmetic mean of the poly(A) tail length was averaged for all genes located on each chromosome.

### Analysis of the GC and GC3 Contents of Gene Coding Regions

A set of human coding sequences (CDS) compiled in a fasta file was obtained from ENSEMBL database (Homo_sapiens.GRCh38.cds.all.fa.gz). All CDS from this file shorter than 60 nucleotides or lacking a start codon were discarded, leaving 83,805 CDS (including 2,585 CDS on the X-chromosome). A custom R script was then used to compute the GC and GC3 contents of the retained CDS from each chromosome and calculate the number of CDS analyzed.

### Principal Component Analysis of 3′-untranslated region Sequences

A bed file containing all the human 3′-untranslated region (UTR) coordinates from the GRCh38 release was downloaded from UCSC (the University of California–Santa Cruz) Tables, and the chromosome fasta files from the same assembly were retrieved from the UCSC download page. A custom R script was used to create a scoring matrix as previously described (Stenberg et al. 2005; Philip et al. 2012). Briefly, the 3′-UTR sequences were extracted, and all the words from monomers to hexamers were counted for each chromosome. The word count was then divided by the sequence length for each n-mer. The scoring matrix was normalized by unit variance scaling then subjected to principal component analysis (PCA) using the SIMCA software (Umetrics, Sweden).

### Analysis of UPF1-Depleted BRIC-seq Data

Data generated in an analysis of mRNA half-lives in the presence and absence of UPF1 in HeLa cells (Tani, Imamachi, et al. 2012) were provided by Nobuyoshi Akimitsu. Only genes for which mRNA half-life data had been acquired in BRIC-seq experiments under both control and UPF1-depleted conditions were included in the analysis (10,649 genes in total, including 339 X-chromosomal genes). Average mRNA half-lives of transcripts from each chromosome were calculated, and the differences between them under the two conditions (siUPF1 − siControl) were then calculated and plotted.

### Statistical Analysis

All statistical analysis and plotting were performed using Statsoft Statistica 12, custom R scripts, SIMCA (Umetrics, Sweden), or Microsoft Excel 2013.

## Results

### X-Chromosome Transcripts Have Significantly Longer Half-Lives than Autosomal Transcripts in Humans

**Fig. 1.—:**
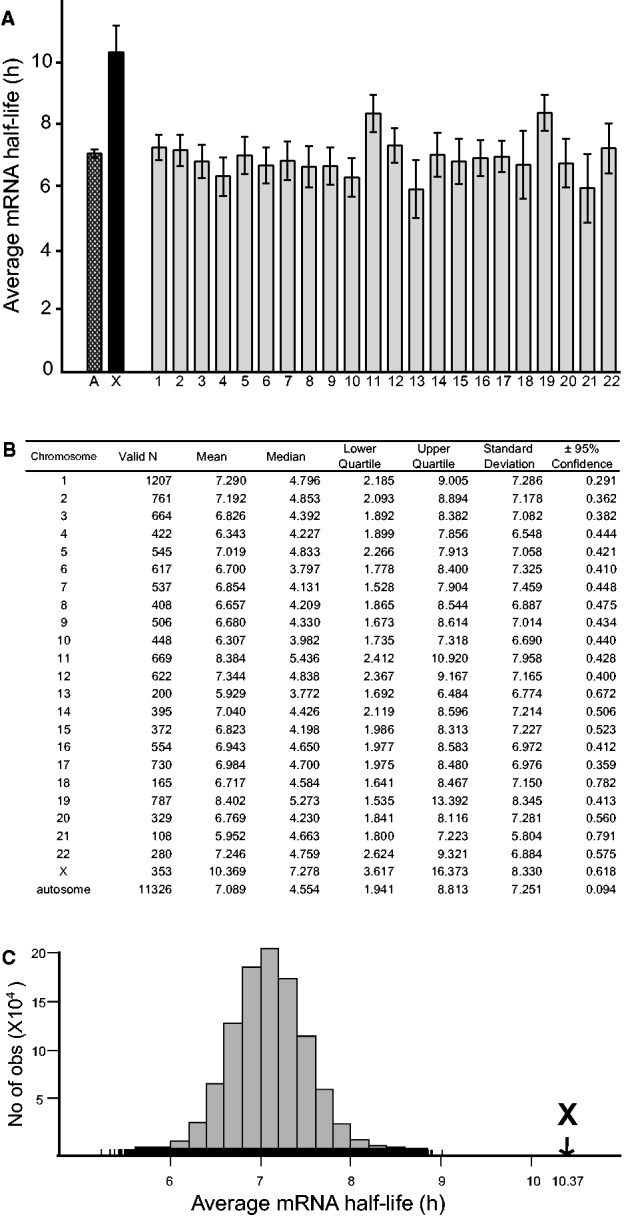
Transcripts of X-chromosome genes have significantly longer half-lives than autosomal transcripts in HeLa cells. (*A*) Average half-life of transcripts of all genes on each chromosome (*n*_X_ = 353, *n*_A_ = 11,326). *A*, average half-life of transcripts of all autosomes. *X*, average half-life of transcripts of the X-chromosome. Error bars indicate 95% confidence intervals. (*B*) Descriptive statistics for the HeLa cells data set. (*C*) Histogram showing the frequency distribution of the averages from 10^6^ samplings of 353 autosomal genes. The arrow indicates the value obtained for the X-chromosome.

To compare the stability of X-chromosome and autosomal transcripts, we first examined the global average stability of mRNA expressed from each chromosome in HeLa cells using published BRIC-seq data (Tani, Imamachi, et al. 2012). In the cited study, mRNA half-lives were calculated and correlated with expression levels in the presence and absence of UPF1, an RNA binding protein, and major component of the nonsense-mediated decay (NMD) pathway, to determine whether known UPF1 targets were direct or indirect and to identify new targets. Our analysis showed that the average half-life of X-chromosome transcripts was 46% higher than that of autosomal transcripts (10.37 and 7.09 h, respectively; fig. 1*A* and *B*), a significant difference (*P* < 10^−^^10^ according to a Kruskal–Wallis analysis of variance [ANOVA] two-tailed test, corrected for number of comparisons; supplementary table S1, Supplementary Material online). In contrast, there were no significant differences between autosomes in this respect, except that transcripts of chromosomes 11 and 19 had somewhat longer half-lives than those of chromosomes 3, 4, 6, 7, 9, 10, and 13 (fig. 1*A* and *B*).

**Fig. 2.—:**
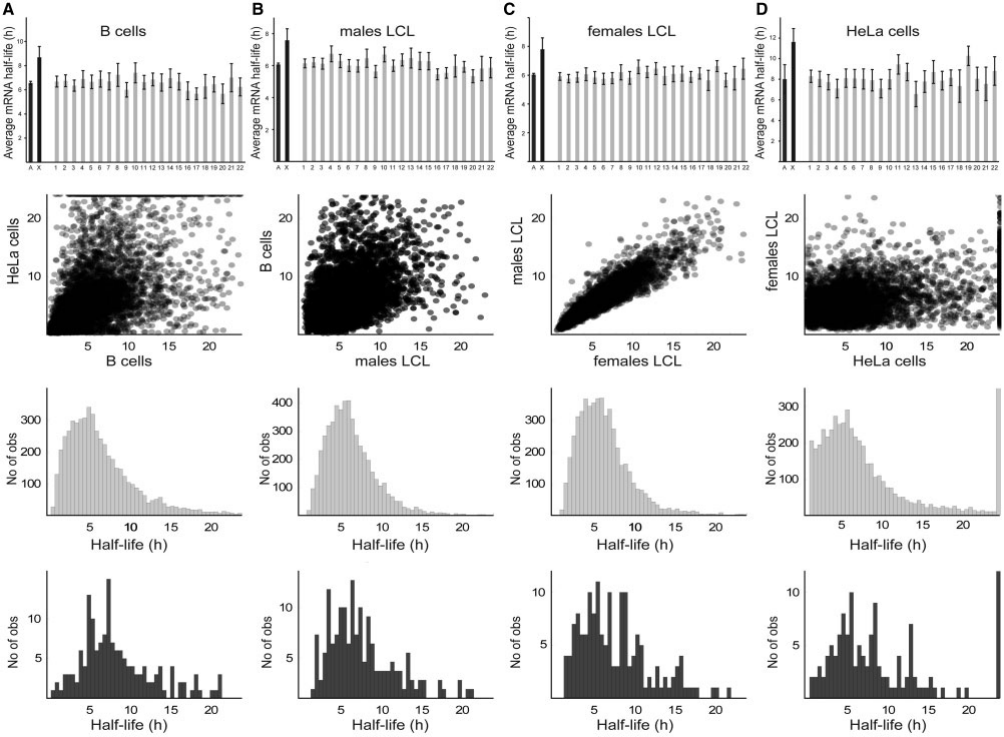
Transcripts of X-chromosome genes have significantly longer half-lives (as measured by several methods) than autosomal transcripts in various cell types and both sexes. (*row 1*) Average half-life of transcripts of each chromosome. Error bars indicate 95% confidence intervals. (*row 2*) Correlation scatterplots of gene mRNA half-life (*h*). Each dot represents one gene. (*row 3*) Distribution of autosomal transcripts’ half-lives. (*row 4*) Distribution of X-chromosome transcripts’ half-lives. (*A*) B cells. (*B*) Gene-by-gene averages in four different male LCLs. (*C*) Gene-by-gene averages in three different female LCLs. (*D*) HeLa cells.

To eliminate the possibility that the higher apparent stability of the X-chromosome transcripts could be due to analysis of a biased subset of genes, we sampled sets of 353 autosomal genes 1 million times and plotted the frequency distribution of their average half-lives (fig. 1*C*). None of these 10^6^ samplings reached the 10.37-h value observed for the studied X-chromosome genes. To exclude the possibility that differential RNA stability could be a HeLa cell-specific phenomenon (due, for instance, to its skewed karyotype), we generalized our analysis by comparing several available data sets, generated from analyses of various cell types (male and female) using different methods (fig. 2). To compare male and female LCLs, we used mRNA half-life data obtained using 4sU pulse-labeling of nascent RNA followed by microarray hybridization (Duan et al. 2013) (fig. 2*B* and *C*). The cited study evaluated interindividual differences in RNA stability and found that these differences are common and contribute to 37% of the observed gene expression differences between individuals. Our analysis of this data set confirmed the higher stability of X-chromosome transcripts, relative to autosomal transcripts, and shows that it occurs in both males and females. This is consistent with Ohno’s hypothesis because both males and females have one functional X-chromosome (caused by X-inactivation in females) but a diploid set of autosomes. Higher X-chromosome transcript stability was also observed in human B cells (fig. 2*A*), using data from Friedel et al. (2009), who identified key regulatory subunits of protein complexes by their higher turnover rates. To compare the data sets, we generated correlation scatterplots (fig. 2), which show low correlation between different data sets, except for the male and female LCLs. Finally, to determine the nature of the differential RNA stability observed (e.g., whether a small subset of X-chromosome genes are highly stable, which would result in a bimodal distribution, or the stability of the whole X-chromosome gene pool is increased), we plotted distributions of half-lives of transcripts from the X-chromosome and autosomes separately (fig. 2*A–D*). When plotted as a distribution, there were too few X-linked genes to evaluate the two models’ validity; therefore, both hypotheses remain plausible (supplementary table S2, Supplementary Material online). Similarly, it was not possible to study genes belonging to the pseudoautosomal region of the X- and Y-chromosome due to their small number and missing data.

### Chromosomal Average mRNA Levels and mRNA Lengths Correlate with RNA Stability for Autosomes Only

**Fig. 3.—:**
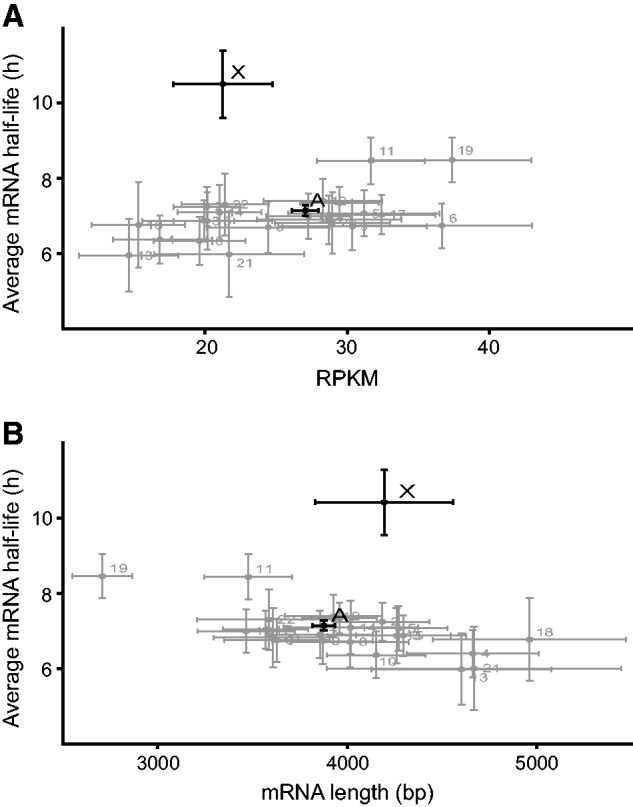
Transcripts of X-chromosome have similar levels and lengths to autosomal transcripts, but significantly different half-lives. (*A*) Average half-life of transcripts expressed by each chromosome versus average steady-state mRNA levels (RPKM). Note the high variability in steady-state mRNA levels in contrast to the significantly higher stability of X-chromosome transcripts. (*B*) Average half-life of transcripts expressed by each chromosome versus average chromosomal mRNA length (bp). Error bars indicate 95% confidence intervals.

We next evaluated whether mRNA stability is influenced by expression levels and/or transcript lengths, which could potentially explain the higher observed stability of the X-chromosome transcripts. For this purpose, we plotted the mRNA lengths and mRNA expression levels versus stability in the BRIC-seq data set (Tani, Imamachi, et al. 2012). We detected a slight overall positive correlation between mRNA half-life and steady state mRNA levels measured as RPKM values (Spearman Rank Order Correlation: 0.28 and 0.36 for X-chromosome and autosomes, respectively) (fig. 3*A* and supplementary table S3, Supplementary Material online). In addition, there was a negative correlation between mRNA half-life and mRNA length (Spearman Rank Order Correlation: −0.30 and −0.23 for X-chromosome and autosomes, respectively) (fig. 3*B* and supplementary table S3, Supplementary Material online). These correlations are consistent with previous findings (Feng and Niu 2007; Geisberg et al. 2014), but do not explain the differences in stability observed between X-chromosome and autosomal transcripts. Notably, the average steady state transcript level (RPKM) differed substantially between individual autosomes (e.g., 2.6-fold between chromosomes 13 and 19), but there was no significant difference in average RPKM between X-chromosome and autosomal transcripts (Kruskal–Wallis ANOVA two-tailed test, *P* > 0.05; supplementary table S4, Supplementary Material online). Importantly, there was a significant difference between the stability of X-chromosome and autosomal transcripts (supplementary table S1, Supplementary Material online), but not in their RPKM values (supplementary table S4, Supplementary Material online). The greatest difference in average transcript half-life among autosomes was only 1.4-fold (between chromosomes 13 and 19), whereas the longest average mRNA half-life for any autosome was still significantly lower than that of the X-chromosome.

### The Higher Stability of X-Chromosome Transcripts Is Conserved between Humans and Mice

**Fig. 4.—:**
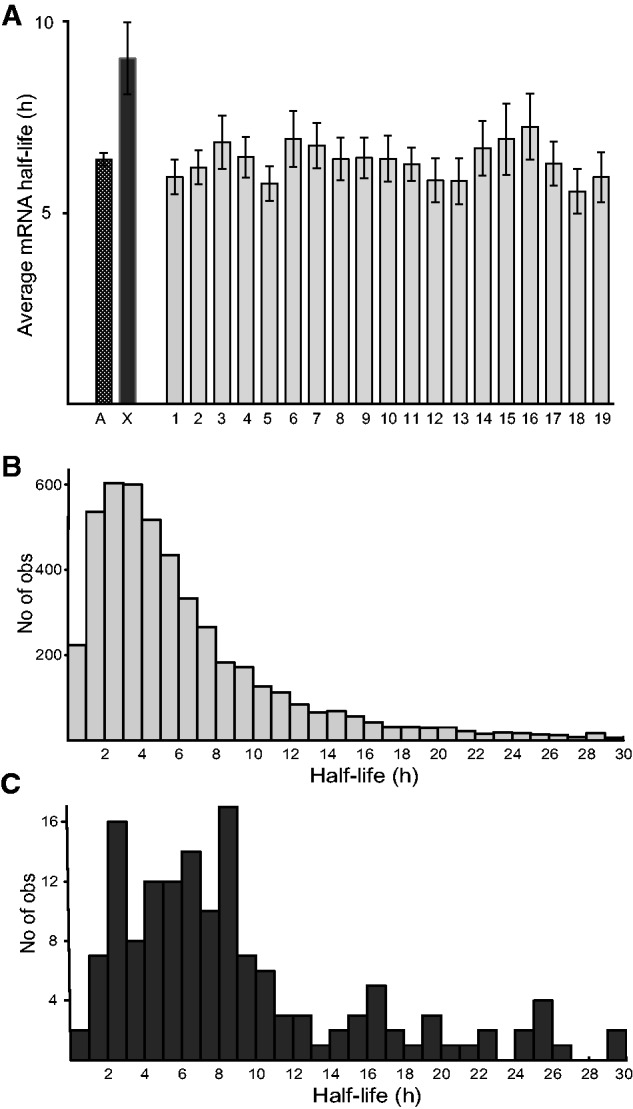
Differential mRNA stability between X-chromosome and autosomal transcripts is conserved in mice. (*A*) Average half-life of transcripts of all genes on each chromosome in murine fibroblasts (*n*_X_ = 151, *n*_A_ = 4,722). *A*, average half-life of transcripts of all autosomes. *X*, average half-life of X-chromosome transcripts. Error bars indicate 95% confidence intervals. (*B*) Frequency distribution of autosomal transcript half-lives. (*C*) Frequency distribution of X-chromosome transcripts half-lives.

Next, we analyzed a data set showing the stability of transcripts in murine fibroblasts (Friedel et al. 2009) to assess the possibility that differential mRNA stability is evolutionarily conserved. The analysis revealed that X-chromosome transcripts are also more stable, on average, than autosomal transcripts in mice (fig. 4*A*) (Kruskal–Wallis ANOVA two-tailed test, *P* < 10^−^^8^). Furthermore, mean half-lives of transcripts from every individual autosome except chromosomes 6, 14, and 16 were significantly shorter than the mean half-life of the X-chromosome transcripts (supplementary table S5, Supplementary Material online). The frequency distributions of half-lives of transcripts of genes along the autosomes and X-chromosome are also similar to the distributions observed in human data sets (fig. 4*B* and *C*).

### Average Ribosome Density Is Significantly Higher on X-Chromosome Transcripts in Both Humans and Mice

**Fig. 5.—:**
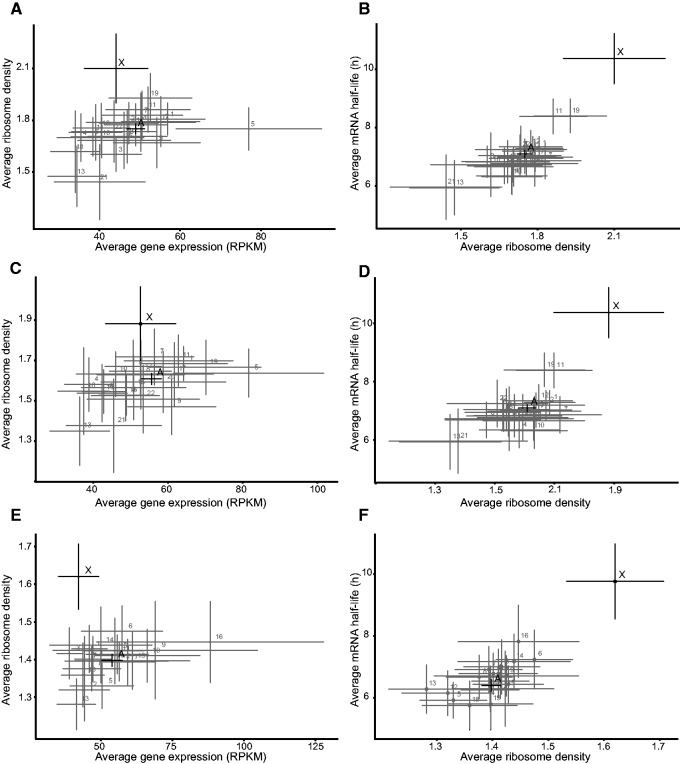
The ribosome density is significantly higher on X-chromosome transcripts and correlates with mRNA half-life but not mRNA levels, in humans and mice. (*A*) Average level of gene expression versus average ribosome density of transcripts of each chromosome in HeLa cells (12 h after mock transfection). (*B*) Average ribosome density versus average half-life (BRIC-seq) of transcripts of each chromosome in HeLa cells (12 h after mock transfection). (*C*) Average level of gene expression versus average ribosome density of transcripts of each chromosome in HeLa cells (32 h after mock transfection). (*D*) Average ribosome density versus average half-life (BRIC-seq) of transcripts of each chromosome in HeLa cells (32 h after mock transfection). (*E*) Average level of gene expression versus average ribosome density of transcript of each chromosome in mouse neutrophil cells. (*F*) Average ribosome density of transcripts of each chromosome in mouse neutrophil cells versus their average half-lives (BRIC-seq) in mouse fibroblast cells. *A*, average for all autosomes’ transcripts. *X*, averages for the X-chromosome’s transcripts. Error bars indicate 95% confidence intervals.

To compare the ribosome density and thus translational potential of X-chromosome and autosomal transcripts, we analyzed ribo-seq data coupled with RNA-seq data obtained from analyses of HeLa cells in a study of the effects of micro-RNAs (miRNAs) on protein production of their targets (Guo et al. 2010). We filtered the data using cutoffs for low read counts to eliminate the ribosome density bias on poly(A) minus transcripts created by use of poly(A) selection in the RNA-seq protocol, but not in the ribo-seq protocol. Our results show that the average ribosome density was higher on X-chromosome transcripts than on the autosome transcripts, both at 12 h (fig. 5*A*) and at 32 h (fig. 5*C*) after a mock transfection of HeLa cells, and in mouse neutrophils (fig. 5*E*). Pairwise Kruskal–Wallis rank sum test of differences between X-chromosome and autosome ribosome densities gave *P* values of 7.303 × 10^−^^5^, 1.358 × 10^−^^3^, and 2.683 × 10^−^^7^ for HeLa cells (mock 12 h), HeLa cells (mock 32 h), and mouse neutrophils, respectively. The more stringent Kruskal–Wallis ANOVA two-tailed multiple comparisons test gave *P* values of 0.0201, 0.375, and 5.6 × 10^−^^5^, respectively (supplementary tables S6–S8, Supplementary Material online). In addition, the ribosome densities correlate well with the calculated average mRNA half-lives from the BRIC-seq analysis and values of both variables are extreme for X-chromosomes of both HeLa cells (fig. 5*B* and *D*) and mouse cells (fig. 5*F*). It should be noted that the mouse mRNA stability and ribosome density data were obtained from analyses of mouse fibroblasts and neutrophils, respectively. Nevertheless, the results indicate that in all considered cases ribosome densities are significantly higher on the X-chromosome transcripts than on autosomal transcripts. Although the average ribosome density correlates with the average half-life of transcripts from all autosomes, we do not observe a strong correlation at the individual gene level (supplementary fig. S1, Supplementary Material online). We conclude that a higher ribosome density on the X-chromosome transcripts may contribute (through effects on both translation rates and RNA stability) to dosage compensation.

### mRNA Half-Life Does Not Correlate with Poly(A) Tail Length, GC and GC3 Contents or any Detected 3′-UTR Sequence Features

**Fig. 6.—:**
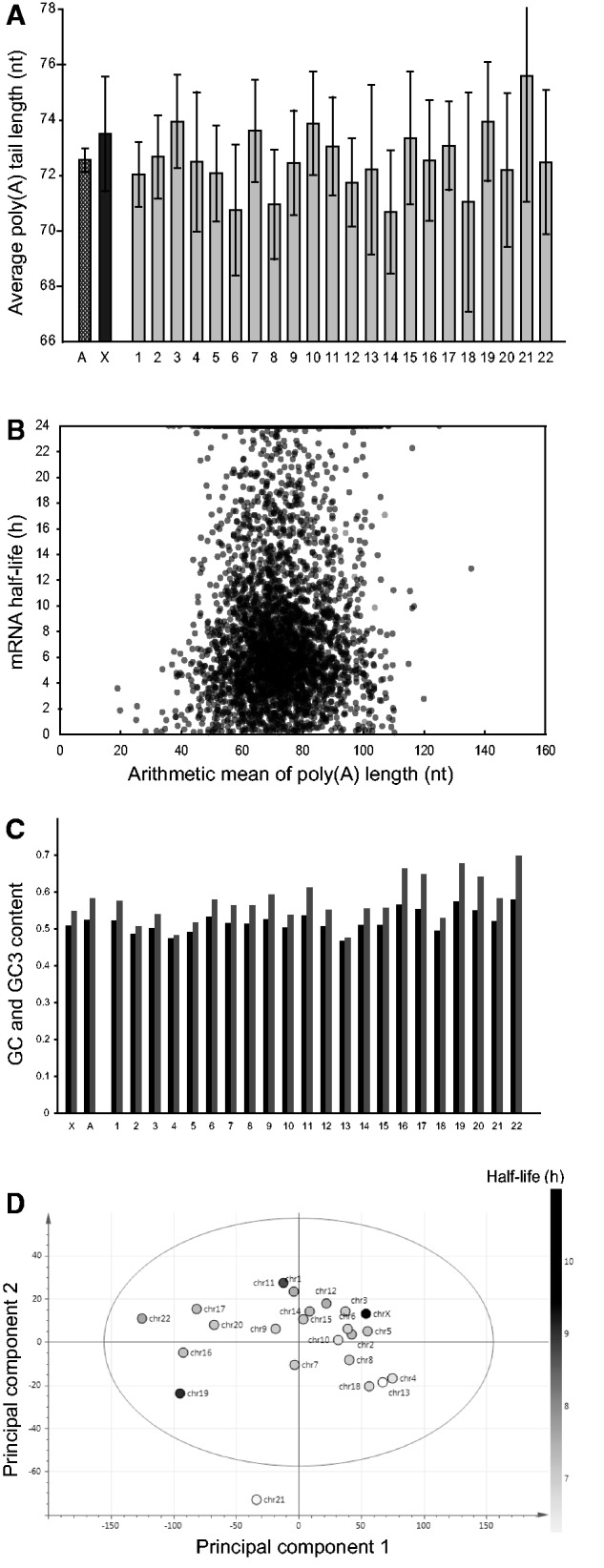
Intrinsic characteristics of mRNAs. (*A*) Average poly(A) tail length of transcripts of each chromosome. *A*, average poly(A) tail length of transcripts of all autosomes. *X*, average poly(A) tail length of transcripts of the X-chromosome. Error bars indicate 95% confidence intervals. (*B*) Gene scatterplot of poly(A) tail length versus half-life. Each dot represents one gene. (*C*) Chromosomal average of GC and GC3 contents of all CDS of the human genome (black and gray bars, respectively): 86,390 in total, including 2,585 situated on the X-chromosome. (*D*) Position of each chromosome along the first and second principal components obtained from a PCA of the 3′-UTR sequences of human genes.

In a search for a mechanism that may potentially increase the stability of mRNA transcripts, we examined intrinsic characteristics of the RNAs, including poly(A) tail length, GC, and GC3 contents of the coding region, and selected 3′-UTR sequence features. A prevailing view is that poly(A) tail length is important for mRNA stability and that poly(A) tail shortening from a certain starting point acts as a timer for mRNA stability (Jacobson and Peltz 1996; Eckmann et al. 2011). We therefore examined whether poly(A) tail length correlates with our studied RNA stability data and, more specifically, whether X-chromosome and autosome transcripts differ in this respect. For this study, we used data acquired from analyses of HeLa cells by Chang et al. (2014) using their newly developed method called TAIL-seq with genome-wide measurements of steady-state poly(A) tail length. Average poly(A) tail lengths of transcripts calculated using these data were very similar for every chromosome including the X-chromosome, ranging from 70 to 75 nucleotides (fig. 6*A*). Although Chang et al. (2014) reportedly detected a weak correlation between poly(A) tail length and RNA stability, as measured by Schwanhausser et al. (2011), we observed no correlation using the HeLa cell BRIC-seq data (fig. 6*B*). We therefore exclude the hypothesis that steady-state poly(A) tail length may be a substantial contributor to the increased observed stability of X-chromosome mRNAs.

The GC content of a gene region can change the physical properties of the DNA, notably high GC contents are associated with high thermostability and a high bendability of DNA molecules (Vinogradov 2003), which could be related to active transcription. Accordingly, for instance, *Hsp70*, *GFP,* and *IL2* mRNA with increased GC contents reportedly have increased expression levels when transfected into human cells (Kudla et al. 2006). The GC3 content represents the GC content of the third positions of codons, also known as wobble positions because specific tRNA species can recognize multiple codons with variations in the third ribonucleotide, so mutations in this position are silent more often than mutations in the other two positions. It has been found to be strongly negatively correlated with genic CpG methylation (Tatarinova et al. 2013). As GC and GC3 content are both associated with gene expression, we calculated them for coding sequences of each autosome and compared averages with average values for the X-chromosome to check correlations with mRNA stability (fig. 6*C*). No significant between chromosome difference of these variables was detected.

The 3′-UTRs of mRNAs are obvious potential sites for elements involved in regulation of mRNA stability because they can affect the fate of an mRNA without altering the protein-coding sequence and may harbor potential protection sequences from 3′-end degradation. Therefore, we subjected all 3′-UTR DNA word frequencies (all words from monomers to hexamers) from each chromosome to PCA, which reduces the dimensionality of data sets and facilitates detection of patterns by segregating observations along linear vectors (principal components or “latent variables”). This approach has been previously used to differentiate three *Drosophila* species’ X-chromosomes and F-elements from the rest of the genome (Stenberg et al. 2005; Philip et al. 2012). The separation of the chromosomes along the first and second principal components, which explain 61% and 8% of the total and remaining variability, respectively, does not separate the X-chromosome from the autosomes (R2 = 0.694, Q2 = 0.58) (fig. 6*D*). Furthermore, there appears to be no general correlation between 3′-UTR sequence composition and mRNA stability (average half-lives, indicated on a linear gray scale; fig. 6*D*).

### Average Stability of Chromosomes’ Transcripts Does Not Depend on a Specific Gene Content or Spatial Organization

**Fig. 7.—:**
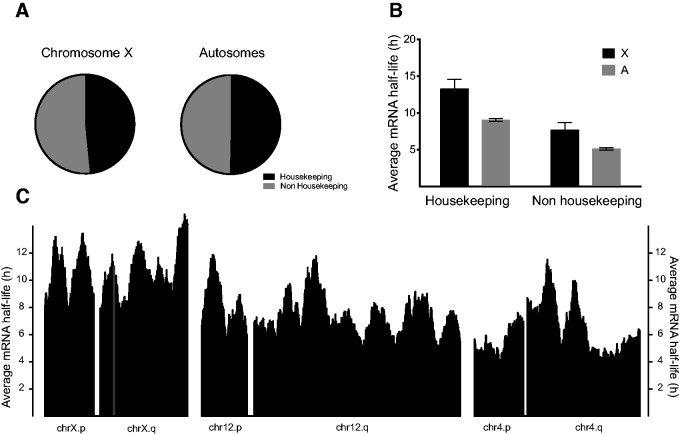
The higher stability of X-chromosome transcripts is not related to the chromosome’s housekeeping gene content or spatial distribution. (*A*) Percentages of housekeeping (black) and nonhousekeeping genes (gray) located on the X-chromosome and autosomes in HeLa cells (*n*_X_ = 353, *n*_A_ = 11,326). (*B*) Average half-lives of transcripts of housekeeping and nonhousekeeping genes on the X-chromosome (black) and autosomes (gray) (*n*_X_ = 353, *n*_A_ = 11,326). Error bars indicate 95% confidence intervals. (*C*) Average half-lives of transcripts of spatial clusters of a sliding window of 30 genes. The bin where *Xist* is located is indicated in gray.

We next asked whether chromosomal organization characteristics could influence the observed average mRNA half-lives. Generally, transcripts of housekeeping genes are more stable than transcripts of regulatory genes, which must be rapidly turned over to allow adaptations to changing conditions (Tani, Mizutani, et al. 2012). Thus, we checked whether the X-chromosome had higher proportions of housekeeping genes than the autosomes, according to the data used in our analysis, as this could potentially explain the higher average stability of X-chromosome transcripts. We divided all genes into housekeeping and nonhousekeeping genes, according to previously published classifications (Zhu et al. 2008), and checked their respective proportions on the X-chromosome and autosomes (fig. 7*A*). Using this classification, the housekeeping to regulatory gene ratio was very close to 1 for both the X-chromosome and autosomes. The ratio can vary depending on the criteria used to define housekeeping genes, but the X-chromosome and autosome ratios remained very similar. Moreover, average half-lives were longer for transcripts of both housekeeping and nonhousekeeping genes situated on the X-chromosome (fig. 7*B*), suggesting the presence of a mechanism that increases their stability independently of gene function.

X-chromosome inactivation is a process that starts locally around the *Xic* and then spreads in *cis*. We hypothesized that the mechanism regulating RNA stability may have coevolved and may therefore operate in a similar spatial fashion. Thus, we checked whether genes with different ranges of RNA stabilities are spatially clustered on chromosomes. We computed the average mRNA half-life in 30-gene sliding windows along each human chromosome arm and plotted all the mRNA stability profiles (supplementary fig. S2, Supplementary Material online) using the BRIC-seq data. The mRNA stability profiles of the X-chromosome and two representative autosomes (chromosomes 12 and 4) are depicted in figure 7*C*. There was no obvious difference in the spatial pattern around the *Xic *(yellow bar on the q arm of chromosome X) and the rest of either the autosomes or X-chromosome. Furthermore, a submission of all X-chromosome genes to Gene Ontology David analysis against the human genome background did not reveal any particular category enrichment on the X-chromosome.

### The NMD Machinery Could Contribute to Higher Stability of X-Chromosome Transcripts

**Fig. 8.—:**
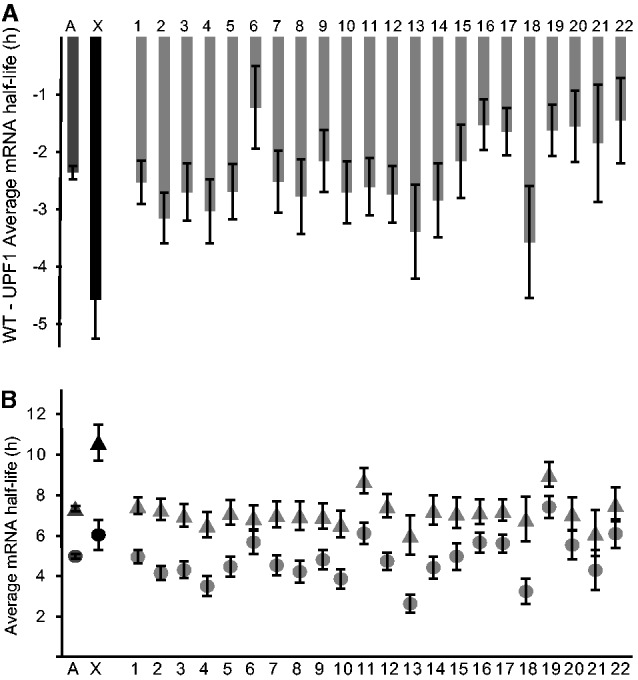
UPF1 knock-down has more effect on X-chromosome transcripts than autosomal transcripts. (*A*) Differences among chromosomes in average half-lives of transcripts after UPF1 depletion. (*B*) Average half-lives of transcripts of each chromosome, before (triangles) and after (circles) UPF1 depletion. *A* and *X*, averages for autosomal transcripts and X-chromosome transcripts, respectively.

It has been proposed that the NMD pathway may participate in dosage compensation, because there is a lower percentage of NMD targets on the X-chromosome than on autosomes in humans and mouse (Yin, Deng, et al. 2009). In addition, the NMD pathway does not exclusively target transcripts with premature stop codons but also functional transcripts in a regulatory fashion, as reviewed by Isken and Maquat (2008). Moreover, Yin, Deng, et al. (2009) showed that depletion of UPF1, a core protein in the NMD complex, decreases the X:AA mRNA steady-state expression ratio in HeLa cells. Given that the known function of the NMD pathway primarily affects the stability of RNAs, we explored potential differences in decay rates of X-chromosome and autosome transcripts following NMD inactivation, using the UPF1 RNAi BRIC-seq data generated by Tani, Imamachi, et al. (2012). Their data led to a surprising observation: Global mRNA stability is dramatically decreased in UPF1-depleted cells (from 8.1 to 5.3 h), contrary to expectations as NMD targets cannot be efficiently degraded in these cells. The reason for this remains elusive, but a possibility is that UPF1 may have a dual role. UPF1 is known to be involved in RNA degradation pathways for some specific target genes, but it could also be a universal stabilizing protein. To test this hypothesis, we plotted the difference in average mRNA half-lives of HeLa autosomes and X-chromosome transcripts before and after UPF1 depletion (fig. 8*A*) and their individual average half-lives (fig. 8*B*). The results show that UPF1 depletion significantly increases average half-lives of transcripts of every chromosome, but the effect is strongest on the X-chromosome. Therefore, UPF1 could be indirectly involved in the maintenance of higher stability of X-chromosome transcripts.

## Discussion

The aims of this study were to explore the potential contributions of two novel elements (differential mRNA stability and ribosomal density) to dosage compensation of the X-chromosome in mammals. To test the hypothesis that dosage compensation could be partially mediated by differential RNA stability, we compared the half-lives of X-chromosome and autosome transcripts in male and female human cell lines of various origins and a mouse fibroblast cell line. For this, we used previously acquired data sets generated by nondestructive labeling methods, which provide more accurate estimates of transcripts’ half-lives and disturb cell physiology less than methods involving blockage of transcription (Friedel et al. 2009). Our results suggest that differential mRNA stability and translation rates of the autosomes and sex chromosomes are important elements of the X-chromosome dosage compensation system and appear to be evolutionarily conserved in mammals.

### X-Chromosome Transcripts Have Significantly Longer Half-Lives than Autosomal Transcripts in Humans

A significantly longer average half-life of X-chromosome transcripts was observed in all the data sets we analyzed (figs. 1, 2, and 4), regardless of the method used, the sex of the material and the cell line. The gene-by-gene correlation between data sets is only strong for the male and female LCLs, which could be due to differences in experimental procedures, cell types, and/or interindividual differences. However, this strengthens our observations because in all data sets, despite rather high global variability, X-chromosome transcripts are significantly more stable than autosomal transcripts. Together, these plots allow us to spot some technical differences and limitations of the methods used. For instance, the BRIC-seq method did not provide data for half-lives longer than 24 h, whereas the other methods could (not all data points are shown, due to the scaling), which affects the distribution of this data set. We conclude that the higher stability of X-chromosome transcripts is not sex-specific, that it is independent of the cell type and technique used to determine the stability, and that it is probably driven by an X-chromosome-specific effect in both sexes.

### Chromosomal Average mRNA Levels and Lengths Correlate with RNA Stability for Autosomes Only

Our analysis shows that the interchromosomal variability in average steady state transcript levels is higher than the 2-fold upregulation of X-chromosome transcripts predicted by Ohno’s hypothesis. Thus, the X:AA expression ratio may not be sufficiently informative to determine whether dosage compensation occurs. However, the chromosomal average mRNA half-life range is much narrower, and there is a statistically significant difference between the autosomes and the X-chromosome in this respect. Additionally, the average mRNA expression levels only correlate with average mRNA half-lives for autosomes. If we assume that the expression output from X-chromosome genes doubled during degeneration of the Y-chromosome as a consequence of a global 2-fold stabilization of the transcripts, we can estimate average values of 5.2 and 10.7 h, respectively, for mRNA half-life and RPKM values before formation of the sex chromosomes. These hypothetical “proto-X” values fit the regression line between RPKM and mRNA half-life for all autosomes well. Therefore, we hypothesize that increases in the half-lives of X-chromosome transcripts raise their levels and thus participate in dosage compensation.

### The Higher Stability of X-Chromosome Transcripts Is Conserved between Humans and Mice

A previous comparison of mRNA stability between mouse and human orthologs found a high degree of correlation (Friedel et al. 2009), suggesting the presence of conserved mechanisms in mRNA stability control. In addition, here we show that in both species, the X-chromosome transcripts are significantly more stable. This finding indicates that mRNA stability has been modulated during evolution of the sex chromosomes as part of a dosage compensatory mechanism. Stabilization of transcripts could also reduce stochastic variation in expression (Yin, Wang, et al. 2009), which may also contribute to dosage compensation. It would be highly interesting to test the validity of this finding for other more distantly related species, and species with a different sex chromosome system.

### Average Ribosome Density Is Significantly Higher on X-Chromosome Transcripts in Both Humans and Mice

Our analysis using ribo-seq data from Guo et al. (2010) shows that ribosome density is generally higher on X-chromosome transcripts than on autosomal transcripts, suggesting that they are more efficiently translated, thereby also contributing to dosage compensation. Furthermore, average chromosomal transcript half-lives and ribosome densities correlate well in both HeLa cells and mouse. These findings strongly indicate that active translation stabilizes transcripts. Thus, we propose that the higher ribosome density participates in dosage compensation by both stabilizing the X-chromosome transcripts and translating more encoded proteins. Because the higher ribosome density was observed both in humans and mice, it is tempting to speculate that an X-specific translational control system evolved in response to pressures to compensate for the degeneration of the Y-chromosome. It remains to be determined whether the increased ribosome density alone explains the observed increase in mRNA stability or whether these are additive effects.

### mRNA Half-Life Does not Correlate with Poly(A) Tail Length, GC and GC3 Contents or any Detected 3′-UTR Sequence Features

None of the studied intrinsic characteristics of mRNAs could explain the observed differences in stability of X-chromosome and autosomal mRNAs. However, we can still imagine a mechanism that would retard deadenylation rates of X-chromosome transcripts to increase their stability, but this is technically challenging to verify genome-wide. RNA modification could also potentially be involved. Our analysis of recently published data sets of possible markers, for example, 5-methylcytosine (Squires et al. 2012; Hussain et al. 2013), 6-methyladenosine (Dominissini et al. 2012), adenosine to inosine (Sakurai et al. 2010), and pseudouridine (Carlile et al. 2014; Schwartz et al. 2014) has not detected any clear deviation of the X-chromosome from the autosomes, and/or are not sufficiently extensive to draw robust conclusions. A complex miRNA network could also potentially regulate the stability of selected RNAs on the X-chromosome. However, in our analyses of miRNA targets on the X-chromosome using TargetScan (Grimson et al. 2007; Friedman et al. 2009; Garcia et al. 2011) we have not detected any clear interchromosomal differences in miRNA targeting.

### Average Stability of Chromosomes’ Transcripts Does Not Depend on a Specific Gene Content or Spatial Organization

In fruit flies, where the dosage compensation mechanisms are better understood, the transcriptional output of the single male X-chromosome is boosted 2-fold (relative to the autosomes’ output) by a “buffering” system acting on monosomic regions and monosomic chromosomes in general (Stenberg et al. 2009; Zhang et al. 2010; Lundberg et al. 2012) in combination with increased expression mediated by the MSL ribonucleoprotein complex (Hamada et al. 2005; Deng et al. 2009; Prestel et al. 2010; Stenberg and Larsson 2011). The buffering of monosomic regions or chromosomes mainly acts on nonhousekeeping genes (Stenberg et al. 2009). In contrast, the MSL-complex appears to bind mainly to housekeeping genes (Gilfillan et al. 2006), but stimulates the expression of both housekeeping and nonhousekeeping genes (Philip and Stenberg 2013). This suggests, albeit indirectly, that the increased mRNA stability is related to an X-chromosome-specific dosage compensation mechanism rather than a more general buffering of monosomic regions. In the study presented here, no difference in the spatial distribution of transcripts’ half-lives along the X-chromosome and autosomes, relative to *Xic*, was detected. We therefore conclude that mRNA stability is not controlled spatially as a function of distance to *Xic*.

### The NMD Machinery Could Contribute to the Higher RNA Stability of X-Chromosome Transcripts

Although the reason for the global reduction in mRNA stability following UPF1 depletion remains elusive, our results indicate that altering global mRNA stability homeostasis (in this manner at least) suppresses dosage compensation mediated by increases in X-chromosome transcript stability.

Taken together, our findings show that X-chromosome transcripts are more stable and more ribosome dense, and thus more abundantly translated, than autosomal transcripts, in both humans and mice. The higher stability of X-chromosome transcripts appears to be a general property conserved between humans and mice that is independent of both sex and gene function. Our results also suggest that these differences are mediated by conserved mechanisms that have evolved in concert with other dosage compensation mechanisms that collectively maintain global expression levels within fitness-optimizing functional ranges. This seems a highly plausible scenario, because balanced control of gene output requires synergistic control and coordination of all the steps involved in gene regulation (transcriptional, posttranscriptional, translational, and posttranslational). We therefore propose that modulation of mRNA stability and increases in translation rates are evolutionary adaptations that compensate for the imbalance between X-chromosomes and autosomes following degeneration of the Y-chromosome.

## Supplementary Material

Supplementary tables S1–S8 and figures S1 and S2 are available at *Genome Biology and Evolution* online (http://www.gbe.oxfordjournals.org/).

Supplementary Data
