# Supplementary material for: Increased Expression of X-Linked Genes in Mammals Is Associated with a Higher Stability of Transcripts and an Increased Ribosome Density
Source: Genome Biol Evol. 2015 Mar 18;7(4):1039–52. doi: 10.1093/gbe/evv054 (PMC4419800; doi:10.1093/gbe/evv054)
Supplement: Supplementary Data [file supp_7_4_1039__index.html]

Increased expression of X-linked genes in mammals is associated with a higher stability of transcripts and an increased ribosome density — Increased Expression of X-Linked Genes in Mammals Is Associated with a Higher Stability of Transcripts and an Increased Ribosome Density — Supplementary Data 

# Increased Expression of X-Linked Genes in Mammals Is Associated with a Higher Stability of Transcripts and an Increased Ribosome Density

## Supplementary Data

files

**Files in this Data Supplement:**

- Supplementary Data - pdf file
- Supplementary Data - pdf file
- Supplementary Data - docx file
- Supplementary Data - xlsx file
