# Supplementary figures and images for: Increased Expression of X-Linked Genes in Mammals Is Associated with a Higher Stability of Transcripts and an Increased Ribosome Density
Source: Genome Biol Evol. 2015 Mar 18;7(4):1039–52. doi: 10.1093/gbe/evv054 (PMC4419800; doi:10.1093/gbe/evv054)

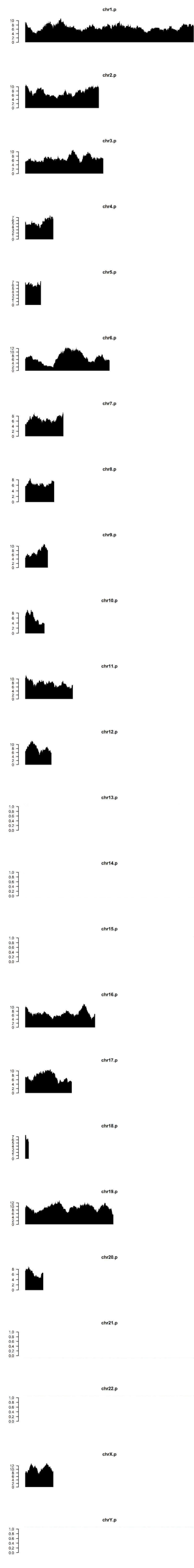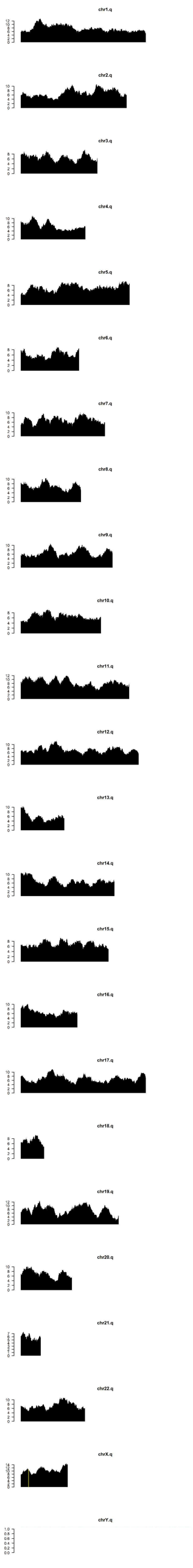

Supplement: Supplementary Data [file supp_evv054_supplementary_fig2_resubmission.pdf]

Supplementary figure 1

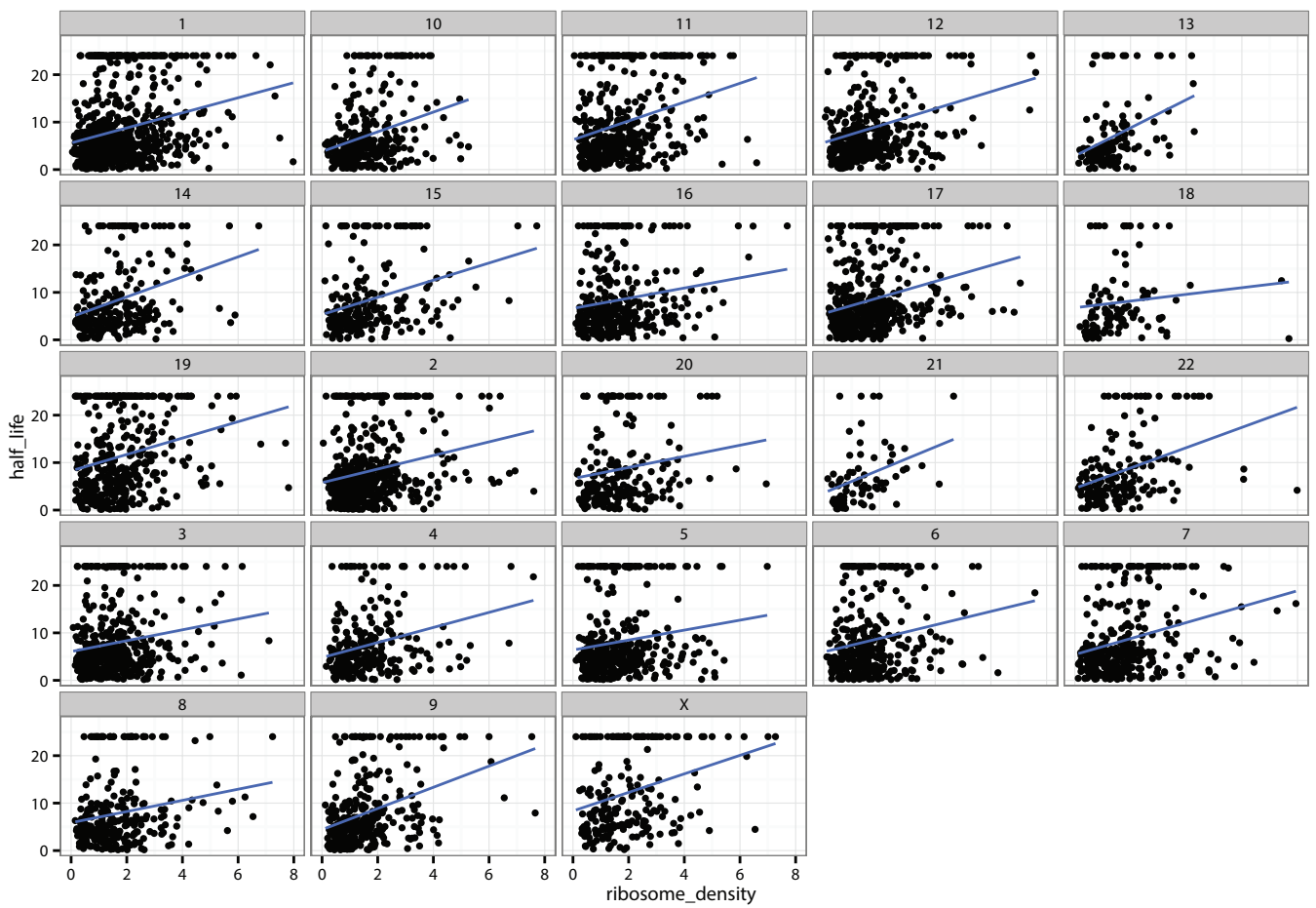

Supplement: Supplementary Data [file supp_evv054_sup_fig1_A4.pdf]
