# Supplementary material for: Increased Expression of X-Linked Genes in Mammals Is Associated with a Higher Stability of Transcripts and an Increased Ribosome Density
Source: Genome Biol Evol. 2015 Mar 18;7(4):1039–52. doi: 10.1093/gbe/evv054 (PMC4419800; doi:10.1093/gbe/evv054)
Supplement: Supplementary Data [file supp_evv054_New_Microsoft_Office_Word_Document.docx]

## Supplementary Fig 1

Single gene scatterplot of ribosome density (12 h after mock transfection) versus half-life (BRIC-seq data) of transcripts of 7301 genes (including 216 situated on the X-chromosome) for which data on both variables were available.

## Supplementary Fig 2

Average half-lives of transcripts of spatial clusters for a sliding window of 30 genes along each human chromosome. The bin where *Xist* is located is indicated in yellow on arm q of the X-chromosome. Chromosome arms for which there were too few data points for this analysis are left blank.

## Supplementary Table S1

Results of the Kruskal-Wallis ANOVA test (*p* values of multiple comparisons) for inter-chromosomal differences in mRNA half-lives for the BRIC-seq sample.

## Supplementary Table S2

Summary statistics of mRNA half-lives for the genes for which data are available in all datasets (B cells, HeLa cells, LCL males, LCL females).

## Supplementary Table S3

Correlation coefficients of exon number, mRNA length, gene expression and RNA half-life between the X-chromosome and the autosomes in the BRIC-seq sample.

## Supplementary Table S4

Results of the Kruskal-Wallis ANOVA test (*p* values of multiple comparisons) for inter-chromosomal differences in mRNA RPKM values for the BRIC-seq sample.

## Supplementary Table S5

Results of the Kruskal-Wallis ANOVA test (*p* values of multiple comparisons) for inter-chromosomal differences in mRNA half-lives for the murine fibroblast sample.

## Supplementary Table S6

Results of the Kruskal-Wallis ANOVA test (*p* values of multiple comparisons) for inter-chromosomal differences in ribosome density for the mock 12 h post mock transfection sample.

## Supplementary Table S7

Results of the Kruskal-Wallis ANOVA statistical test (*p* values of multiple comparisons) for inter-chromosomal differences in ribosome density for the mock 32 h post mock transfection sample.

## Supplementary Table S8

Results of the Kruskal-Wallis ANOVA test (*p* values of multiple comparisons) for inter-chromosomal differences in ribosome density for the mouse neutrophil cell sample.
